# Assessing the Effect of Disturbances on Ectomycorrhiza Diversity

**DOI:** 10.3390/ijerph6020414

**Published:** 2009-02-01

**Authors:** Virgil Iordache, Felicia Gherghel, Erika Kothe

**Affiliations:** 1 Department of Systems Ecology, University of Bucharest, Spl. Independentei 91–95, 050089, Sector 5, Bucuresti, Romania. E-Mails: virgil.iordache@g.unibuc.ro (V.I.); 2 Microbial Phytopathology, Institute of Microbiology, Friedrich Schiller University Jena, Neugasse 25, 07743 Jena, Germany. E-Mails: fgherghel@ice.mpg.de (F.G.)

**Keywords:** Ectomycorrhiza, metals, biodiversity, succession, landscape ecology, ecotoxicology

## Abstract

Ectomycorrhiza (ECM) communities can be described on a species level or on a larger scale at an ecosystem level. Here we show that the species level approach of successional processes in ECM communities is not appropriate for understanding the diversity patterns of ECM communities at contaminated sites. An ecosystem based approach improves predictability since different biotic and abiotic factors are included. However, it still does not take into account the hierarchical structure of the ecosystem. We suggest that diversity patterns of ECMs communities in forests can best be investigated at three levels. This hypothetical approach for investigation can be tested at sites of secondary succession in areas contaminated with metals. Once the diversity patterns are appropriately described by a hierarchical ecosystem approach, to the species level is used to explain these patterns by populational and ecotoxicological mechanisms.

## 1. Introduction

From an applied perspective, the rehabilitation of disturbed ecosystems is a matter of controlling the secondary succession processes [1]. Such a succession perspective has been adopted currently for the rehabilitation of areas contaminated with metals [2, 3]. The study of secondary succession processes in areas contaminated with metals thus offers interesting opportunities for addressing basic research, and at the same time has a clear impact on the remediation of these areas. The purpose of this review is to critically analyze the literature concerning secondary succession processes involving ectomycorrhizae (ECM) in contaminated areas, and to propose an improved theoretical and methodological framework for the research on succesional processes.

In the first part, we review the general context of secondary succession and the role of ECMs (Section 2), then we show the limits of the current approaches (Sections 3.1, 3.2) and, after analyzing the application of the ecosystem approach to fungal succession (Section 3.3), propose an improved, ecosystem based, theoretical framework of analyses (Section 3.4). In the last section we outline the experimental design for the implementation of these theoretical ideas.

## 2. Ectomycorrhizae in Secondary Succession

### 2.1. Ecological Role of Mycorrhizal Fungi in Secondary Succession

By analogy with plant communities, fungal successions have been described as primary, when pioneers have colonized a virgin surface, or secondary after a major event or “disturbance” has shifted the course of a succession [4]. Increased attention is given to the spatial variability of fungal communities in field conditions, and to how these communities evolve as ecological conditions change through disturbances [5]. Small-scale, natural disturbances like severed roots, mixed soil horizons and litter layers, or change of local pH and nutrient availability, are likely to create new habitats for ECM fungi, and thus increase their diversity in forests [6]. On the other hand, soil disturbance can lead to a decrease in the number of morphotypes [7].

In most cases of secondary succession, net ecosystem production is negative immediately following disturbance, and in such cases, output rates can exceed input rates [8]. N mineralization and nitrification generally increase shortly after disturbance, and P availability declines later in soil development. Changes in weathering rates are likely to be lower during secondary succession than during primary succession, given the same climate, parent material, and relief. The fundamental contrast between primary and secondary succession is due to the chemically unstable nature of the fresh parent material in a new site [8].

Mobilization of nutrients and dissolution of minerals are of key importance for plant growth and soil formation, as well as for long term ecosystem sustainability. Weathering is the only natural, long-lasting mechanism by which acid precipitation can be neutralized and nutrients lost via leaching or biomass harvesting can be replenished. Biotic weathering by roots and microorganisms is believed to occur, not only through H^+^ production, but more importantly exudation of complexing ligands [9]. Such ligands include simple organic acids (e.g. oxalate and citrate), siderophores, polyphenolic acids and acidic polysaccharides. Considering the pH of acid forest soils, it is feasible that both hydrogen ions and organic ligands are important. Moreover, the possible effect of organic ligands, both simple compounds and fulvic/humic acids, passively leached from the organic soil, should not be overlooked [10]. Biotic processes are important for the mobilization and biogeochemical cycling of nutrients [8].

The positive role of mycorrhizal symbiosis under stress conditions for plant establishment, growth and nutrition [11] was demonstrated for ericoid mycorrhizas [12], ECM [13, 14] and for arbuscular mycorrhiza [15]. For boreal forest trees belonging to the plant families of *Betulaceae*, *Fagaceae*, *Pinaceae* and *Salicaceae,* the ecologically most important symbiosis is the ECM formed between (mostly) basidiomycete fungi and their host trees. The plants are reported to be able to accumulate or tolerate higher metal concentrations in mycorrhizal symbiosis as compared to non-mycorrhizal plants [16]). Differences in ectomycorrhizal effectiveness for improving tree growth and tree nutrition are often species specific [17], or even strain specific [18, 19]. Most trees show low specificity towards their symbionts, which is they form associations with many different fungi. Some fungi, however, show high host specificity and interact with only one host plant [20]. The expressed metal tolerance of the symbiotic association therefore depends on the mycorrhiza formed in a given ecosystem, and the metal involved [21, 22]. The rate of colonization is determined by biotic (host, fungus) and abiotic (environment, pollution) factors. Mycorrhizal fungi are well known for increasing nutrient uptake, but their effects on soil physical structure and water flow are less well understood [23].

Both the disturbance by clear cutting and disturbance by contamination with metals have been investigated. For instance, changes in fungal species composition were found to be driven by changes in the biology and chemistry of the soil environment after clear cutting as much as they are by loss or change in fungal inoculum [24]. The contamination of an ecosystem can lead to decresed genetic diversity of ECM populations, and even eliminate (or preclude the colonization by) some populations. The genetic variation in the population of *Suillus luteus* from an unpolluted site was considerably larger than that observed at a polluted site [25]. With increasing distance from Zn smelters, the frequency of Zn tolerant genotypes decreases [26]. Addition of small concentration of metals to isolates of *Aspergillus niger* from mine surroundings was even found to stimulate the production of biomass, compared to isolates from not contaminated areas [27].

As a conclusion of the assessment of disturbance as an ecological factor, it can be strated that disturbance is a term too general to allow meaningful correlations with changes in biodiversity. Different types of disturbance lead to different effects on the ECM community structure and thus should be analyzed analytically before trying to assess synergistic effects. A further limitation is that studies after of disturbance are rare [28] and absent, to our knowledge, in case of contaminated areas.

### 2.2. Ectomycorrhiza in Metal-Rich Environments

In many ecosystems the ECM fungi are primary agents of decomposition. The amount and differentiation of the extramatrical mycelium [29] is a very important ecological factor for tree performance, apart from the abundance of ECM [30]. The extramatrical mycelium is a sink for tree carbohydrates transferred to the root system on the one hand, but is also an important functional extension of the root system [31]. This explains why mycorrhizae may act as long distance biofilters for plant uptake under metal stress conditions.

Toxic metals are believed to affect fungal populations by reducing abundance and species diversity and selection for a resistant/tolerant population [32]. Given the broad spectrum of fungi forming ectomycorrhizal associations, it is likely that different fungi may affect metal sensitivity of their hosts by different mechanisms. One, more physical approach, is provided by the mantle formed by fungal hyphae surronding the roots aiding plant growth in soils with high concentrations of heavy metals. Zinc, cadmium and arsenic have been found in high concentrations in cells of the hyphal mantle [33] and it is thought that certain mycorrhizal isolates accumulate and immobilise heavy metals in their fungal mantle. The ectomycorrhizal mantle itself does not have a large contact area with the soil, while the emanating hyphae and rhizomorphs greatly increase the volume of exploited soil [12]. The way in which fungi exploit the surronding soil can be used to describe different functional types. Different exploration types of ectomycorrhizae are distinguished based on the amount of emanating hyphae and the presence and differentiation of rhizomorphs [18]. The transport function of extramatrical mycelia has been well documented [31, 11] and Kammerbauer *et al*. [29] provided evidence of a relationship between the extent of rhizomorph organization and transport rates of phosphate and water by these hyphal structures. Fixed mycorrhizas revealed strong accumulation of Ca, Fe, Zn and Pb within the fungal mantle and in the rhizomorph [34]. Horewer mycological studies are rare in post mining landscapes, and it is necessary to improve our knowledge about the succession of ECM fungi on reclamation sites [7]. In addition, there are not enough comparative studies for building generalizations on the processes by which mycorrhiza establish and persist under varying environmental conditions [5].

## 3. Succesion and Diversity in Ectomycorrhizal Communities

From a system’s ecology perspective, succession is a process occurring at an ecosystem level (community and its environment), so it is not appropriate to search *a priori* for reductive species level understanding of succession, while it is meaningful to look for species level mechanisms supporting succession in a community and ecosystem context. The search for indicator species of successional stages has been, however, a part of the reductive paradigm, both in plant ecology and in fungal ecology. Consequently, much of the literature dealing with ECM succession is dedicated to the concept of early and late succession species, as label species for certain succession stages.

### 3.1. Early- and Late-Stage Species Approach

Mason *et al*. [35, 36] coined the term early- and late-stage fungi for groups of species identified based on basidiocarp production around *Betula* trees. The early and late succesional dominants could be affected by resource availability [37, 38]. Early species are characterized mainly by reproduction by spores, while late species by clonal expansion. Removal of forest floor increased both the fungal species richness and abundance of fruiting bodies, but increase in fruiting body production occurred mainly in early succession fungal species [39]. Large and persistent genets formed by clonal expansion in some ECM species (*Suillus* spp.*, Xerocomus* spp.) were shown to possess stress tolerant adaptive characteristics (mycelial cords or strands) that facilitate their competitive ability in mature forests [40], while for other species (e.g. *Russula* spp.) growth results only from mycelia radiation in multiple directions. Persistence of a genotype of *Russula* over an 11 year sampling period was also found. The clonal behavior may have consequences on the colonization of new trees: disturbing the tree roots of existing plants changed the fungal species that formed mycorrhizas on roots of planted seedlings adjacent to existing plants [39]. In addition the disturbance at the tree root, the distribution of early and late species are influenced by the tree species, by differences in the life cycle of the tree, or by litter type. Early stage species for instance were found with *Quercus* up to 20 years, while with *Betula*, up to 6 years have been described. This apparently correlated with the life-time of the tree species [41]. Different litter types below trees also have been found to induce different ECM communites to develop, linked possibly to functional differences like P cycling [42].

Another influencing factor is the overall environment of the tree. Air pollution, e.g. can influence the nutritional status of the tree and indirectly the quantity of organic exudates available for ECM, leading to unfavorable conditions for late ECM species [41]. And finally, the age of the roots is a biotic determinant of ECM types. Bigg [39] found that the youngest roots were populated with early-succession species, while older parts of the root system were associated with later-stage species. Thus, a habitat separation between early- and late-stage ECM communities are be seen in the same forest, suggesting that processes of ECM succession are either infra-ecosystem (if we accept that the forest is the ecosystem), or that the forest is an assemblage of (micro)ecosystems (if we accept that the fungal community supports a micro-ecosystem).

### 3.2. Criticism of the Early Versus Late-Stage Species Approach

The first direction of criticism provides exceptions to the characteristics shortly mentioned above (1). Another criticism points out the major role of the dispersion and other biological mechanisms in regulating the communities’ structure over succession (2). A third line puts the accent on the influence of the environment on the succession mechanisms (3). An integrative approach is based on an ecological perspective (4).

(1) For instance Fiore-Donno [43] demonstrates that in a mature forest two late stage species have contrasting colonization patterns: one by clonal growth, the other one by sexual spore propagation. Consequently, one can not expect necessarily higher genetic diversity in early-stage communities.

(2) Newton [44] proposed a functional classification of fungi based on epidemiological, dispersion characteristics (the relative ability of different fungi to colonize and spread from different sources of inoculum) in search for more appropriate classification criteria than early and late successional. The different morphs (emanating hyphae or the presence and differentiation of rhizomorphs, mantle type, laticifers, cystidia, sclerotia, the hydrophoby) were used to classified ECM in a wide range of potential exploration groups extending from the contact- to the long-distance exploration types [18, 45]. Mycelia that remain non rhizomorphic are thought to reflect a limited ability to explore surrounding soil, while mycelia that comprise highly differentiated rhizomorphs are regarded as more adapted to long-distance exploration [18]. To some extent, Agerer’s classification is relevant also for the early- versus late-stage classification, as one can expect to have long distance exploration types especially when the nutrients are scarce, i.e. in early stage communities. Buscot *et al*. [5] also suggested that it should be possible to re-classify the species involved into a small number of groups biological and ecological traits were used therein, ranging from dispersal and foraging abilities to stress tolerance and nutrient mobilization and uptake. These parameters for classification rely on “ecological strategies” as described for fungi by Pugh [46].

(3) Keizer and Arnolds [41] studied the relationship between *Quercus* tree age and numbers of ectomycorrhizal species and sporocarps, and found that changes in species composition and diversity showed much variation correlated to different environmental conditions, and also that succession in later stages cannot be explained by root extension alone (after 30 years, the soil was entirely occupied by fine roots). The crucial role of soil factors in the course of succession had been previously suggested [e. g., 47].

(4) From an ecological perspective it became obvious that both environmental variables and dispersal were important factors shaping mycorrhizal communities. This stresses the importance of using a metacommunity approach when dealing with the diversity and succession processes of a certain community [48]. In particular, the distance to other tree islands [49] resulted to be a key factor controlling ECM diversity at tree islands (forest) level.

What can be retained from the early/late-stage distinction is synthesized in table 1 and is well summarized by Keizer and Arnolds [41]: The concepts of early- and late-stage fungi are primarily based on physiological characteristics of species and indeed are useful to unterstand early phases of primary forest succession. However, they are not appropriate to describe ECM succession under field conditions over a longer period since: 1) some early-stage fungi are restricted to young trees but others are maintained on the root systems of old trees; 2) some late-stage fungi appear already with young trees; 3) seedlings near mature trees may be infected by late-stage fungi; 4) late-stage fungi are dominant during some 90–95% of the lifetime of a tree and can be divided into several groups.”. We hypothese an association between exploration types and early – late species (Table 1), having as rationale the fact that early succession environments are usually characterized by small availability of nutrients and C, which could give advantage to medium and long exploration types.

### 3.3. Application of the Ecosystem Approach to Fungal Succession

The ecosystem concept was used to explain the high diversity of ECM communities, and the distribution and dynamic of this diversity: the high diversity of ECM was explained by referring to the concept of niche, fundamental in ecosystem theory. Dickie [51], for instance, points out that “ectomycorrhizal” fungi encounter a highly variable environment with myriad possible niche dimensions. Many of these niche dimensions are relatively narrow in breadth. Nonetheless, dimension breadth is relatively unimportant compared with dimension numbers (*n*), as available niche space in a community, i.e. the ‘*n* dimensional hypervolume’, increases multiplicatively with niche breadth but, exponentially with increasing dimension numbers”.

The differences in ECM diversity from one ecosystem to another in space and time were explained by correlating them to the abiotic characteristics of the ecosystem or by attempting to build an ecosystem level succession theory. This kind of work seems to have started with Christensen [52] who investigated 36 ecosystems and used classification, ordination and regression techniques to describe the species composition of the fungi communities. During the International Biological Program there was a vogue for comparing fungal succession on different types of litter [4]. For the particular case of ECM fungi, Bigg [39] showed, that usually young stands have few, very abundant fungal species, with other species present in low to very low quantities. Over time, the community changes to more species present, but roots still be dominated by relatively few species. So species richness would increase with succession, but the evenness will remain more or less the same. Dighton and Mason [53] had previously developed a three stages model in which species richness increases from young to medium-aged stands, then strongly decreases in old stands, to reach a very low final level (following the vegetation pattern in *Fagus* forests, for instance), apparently contradictory by Bigg’s [39] model. Because both models reflect correct data sets, it seems that there is no unique diversity pattern in the dynamic of ECM with succession. Twieg *et al*. [54], for instance, stated explicitly that simple categories such as ‘early stage’, ‘multi stage’, and ‘late stage’ were insufficient to describe fungal species’ successional patterns and that ECM fungal succession may be best described in the context of stand development, without the need for a universal explanation theory.

From the above short overview it can be seen that, until now, the application of ecosystem concept in the study of ECM diversity patterns in space and time had more a heuristic than a quantitative explanatory value. This situation arose from the fact that the ecosystem approach seems to be applied especially in the interpretation phase of the research programs dealing with ECM, and to a lesser extent in the design phase. This shortcoming can only be rescued by specific design of experiments.

### 3.4. Improved Framework for the Ecosystem Approach

A key issue for ensuring the success of the ecosystem approach is to identify the ecosystem’s structure at the appropriate time and space scale. For instance, if there is one ECM community in a forest, then it is meaningful to estimate ECM richness and diversity directly at a forest level (α diversity), but if there is an assemblage of ECM communities organized on two hierarchical levels, like in a forest which experiences a contamination gradient, then the diversity should be characterized at three levels (α, β and γ). In the first case the succession processes take place directly at forest level. In the second case, the dynamics of diversity at forest level reflects succession processes occurring at tree level (depending on which contamination is present at a single tree), at forest level (like successional stage of the forest) and at ecosystem level (e.g. dispersion mechanisms as controlled by vegetation dynamics and contamination). Thus, both bottom-up (development of single trees at heterogeneous contaminated sites), and constrained top-down control (by meta-community level processes) will be experienced at the same time. This makes the investigation of different levels in an ecosystem approach more useful than restricting the analyses to either bottom-up or top-down models of ecosystem research.

A methodologically relevant definition of the basic unit at which to consider diversity (a development from the elementary community notion) is provided by Pahl-Vostl [55] under the name of ‘trophic-dynamic module’ (TDM). A TDM is defined as the groups of biological populations having 1) similar rates of biomass cycling (inversely correlated with lifetime of the individuals), 2) the same location in space and time, and 3) similar roles for the species in the food web. Application of criterion 1 leads to dynamic classes, further application of criterion 2 leads to dynamic modules, which by criterion 3 are split in TDMs. The above definition can be amended [56] with the remark that some populations can be included in more TDMs at the same time, because of their internal structural diversity. For instance, decidious tree populations have parts with very different rates of biomass cycling, like leaves and wood (criterion 1), as well as parts with different location in space like below *vs*. above ground (criterion 2). Thus, the trees will belong to at least 3 TDMs: 2 above ground and one below ground. The notions of “same order of magnitude”, “same location in space and time”, and “same role in food web” are to be defined by the researcher, and can be applied more stringent or relaxed. In the most stringent application, they will lead to a model identical with the “reality” (isomorphic model). If relaxed too much they will lead to a model too aggregated and having lost the key characteristics of the real system (simplistic model). Only at an appropriate intermediate level, they will lead to a model simple enough for explanatory value, but keeping the basic characteristic of the system (homomorphic model).

### 3.5. Implementation of TDMs

**Figure 1.**
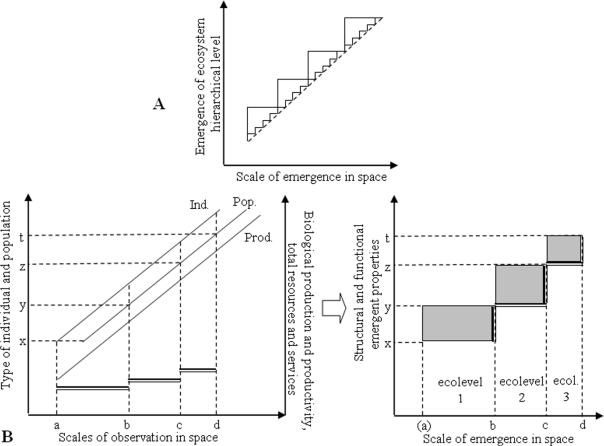
A Simplistic models of the relationship between space-time scale of analyses and the emergence of ecosystem hierarchical levels. The linear model (dotted line) assumes that there is linear appearance of new emergent properties when increasing the scale of analyses, without need to privilege a certain scale (this model is preferred by those considering that ecosystems are methodological concepts applicable at any scale). The nested hierarchy models (continuous line in smaller or larger steps) assume that at certain scales there are jumps of emergent properties allowing the identification of an ecosystem level, then these ecosystem interact over a range of intermediary scales and at other points there is another jump, and so on (such models are preferred by those considering the ecosystems are “real” entities). Note that their can be different nested hierarchy models depending on the privileged scale at which emergent properties are identified. B The relationship between the scale of biological structural elements and processes (individuals, populations, left graph - right axes, production and productivity, left graph - left axes) and the hierarchical structure of ecosystems (right graph). At scales of observation from a to b (corresponding to ecological level 1) one can perceive all types of individuals (and their populations) from x to y, but only some of the individual types from y to z (and not their populations). Then TDMs including populations of type y to z are said to “emerge” at higher hierarchical ecological level 2. Grey areas on the right graph suggest the multidimensional spaces characterizing each ecological level, in which the processes supporting the productivity of each level can be conceptualized. Note that the linear models from the left graph can be cut in a different way leading to alternative hierarchies.

While succession in ecosystems is a process taking place at the level of the networks of TDMs, mechanisms may be analyzed for a group (e.g. fungi) at TDM level. The scale of the TDMs varies hugely, which implies that this is not one “true” scale for ecosystem processes or a simple, nested hierarchy of ecosystems (Figure 1). Rather, emergence of new structural (e.g. new TDMs) and functional (e.g. increase in overall biological productivity, or changes in the rates of biogeochemical processes) properties should be defined and used to drive the mathematical function that links scale and emergence of new properties in different areas and in different periods of time.

As ECM fungi have more or less the same rate of biomass cycling and the same role in food-webs, one cannot expect the separation of ectomycorrhizal TDMs based on these criteria. If species richness of ECM fungi is investigated, islands of trees of similar age and species composiction ranging in size from <10 to >10000 m^2^ show, ECM species richness is significantly reduced on smaller and more isolated tree islands, and the species–area slope that we observe (0.20–0.23) is similar to average slopes reported for macro-organisms. Species occurrence patterns across tree islands and investment trends in fungal fruit bodies suggested that a trade-off between competition and dispersal could play an important role in structuring ECM assemblages [49].

Another impact on TDM separation is seen by sampling effort. Appropriate estimation of diversity was found to be a difficult task because of the large number of samples needed and the heterogeneous distribution of ECM. By constructing species area curves for data published in previous studies, in most cases insufficient sample numbers were analyzed such that diversity of ECM taxa present was not fully covered. Anderson and Cariney [57] show that it is necessary to take cores at least 3 m apart, in order to achieve the greatest sampling efficiency. At the same time they point out that community composition is variable at much finer scale (5–20 cm), with a complete change in ECM community composition occurring in some cases at a scale of 50 cm. In the vertical dimension, different fungi typically occupy different horizons which also needs to be covered by sampling to establish community structures for ECM.

Applying the separation of TDMs to early *versus* late-stage ECM communities at the same tree, TDMs can be distinguished depending on the age of the roots (two TDMs per tree), the net differences in communities structure with depth (humic layer vs. anorganic layer, two TDMs per tree) and the clonal development of late-stage ECM (potentially allowing the same population to occupy more than one tree).

### 3.6. Model for ECM Community Structure Analyses

**Figure 2.**
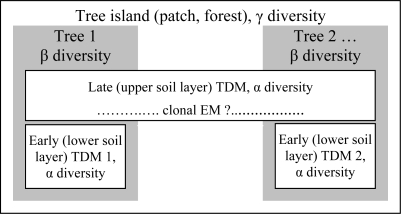
Model of ECMs community structure in a forest: the types of trophic dynamic modules (TDMs) and the structure of ECMs’ diversity. According to the model the diversity of ECMs should be assessed at three hierarchical levels: α diversity at small community level (TDM), β diversity in sets of TDMs around trees, and γ diversity in the set of sets of TDMs, in forest patches.

Based on these considerations, we propose a model of ECMs community structure in a forest (Figure 2). Using this theoretical framework, we are able to identify the following components of ECM structural diversity in a tree island: number of TDMs, species richness inside each TDM (α), at tree (β) and at forest level (γ), and finally evenness inside each TDM.

This way of conceptualizing the structural diversity allows a functional interpretation. For instance, changes in microbial diversity did not always correspond to changes in functional redundancy [58]. The reason for this is that diversity is usually characterized unstructured, at tree island level, which mixes the diversity of different TDMs. As functional redundancy of species occurs only at infra-TDM level, an increase of overall diversity (across pooled TDMs) does not reflect functional redundancy. E.g., decrease of redundancy in one TDM, coupled with an increase in another TDM (or appearance of new TDMs), would lead to similar results. The approach of defining structural diversity, in contrast, allows to quantify the role of each species in the production of ecosystem services by investigating the influence of each species on the rate of relevant processes occurring at functional group (TDM) level [59,60]. At the same time, the extent to which mycorrhizal fungi contribute to the resource partitioning by physiological connections between plants is shown [61].

## 4. Implementing the Improved Framework

**Figure 3.**
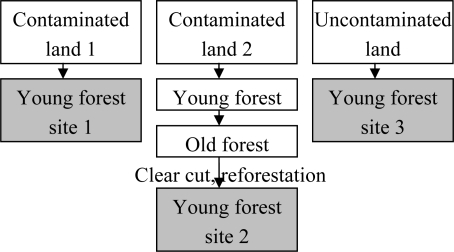
Example for site succession comparison. Each site is investigated at TDM, tree, and forest level and the data are processed (see Figure 4). This set-up allows comparison of contaminated and uncontaminated sites as well as primary and secondary succession, where reforestation established the secondary succession.

In order to make this theoretical ideas approachable, an experimental design should be set up in which 1) different sites are investigated at tree, community and ecosystem level, 2) appropriate data processing procedure is devised, and 3) results are interpreted at all hierarchical levels. Here, we use data of a field study to implement the framework set up in the previous chapters (Figure 3; details see [62]).

We assume that the general succession pattern of ECM communities at TDM level in the first phases is an increase in richness and evenness [53]. The effect of heavy metal contamination would be introduce a selection pressure, especially during early stage mycorrhization. The pedogenesis with the development of an organic soil layer would attenuate this process since the available organic molecules would lead to sequestration of heavy metals. Taken together, we would predict that the richness and evenness of ECM communities at tree level in a secondary succession young forests grown on contaminated land is larger than in a primary succession young forests grown on contaminated soil in the upper TDM (defined here as the upper soil layer), but smaller in the lower TDM (defined as lower soil layers).

**Figure 4.**
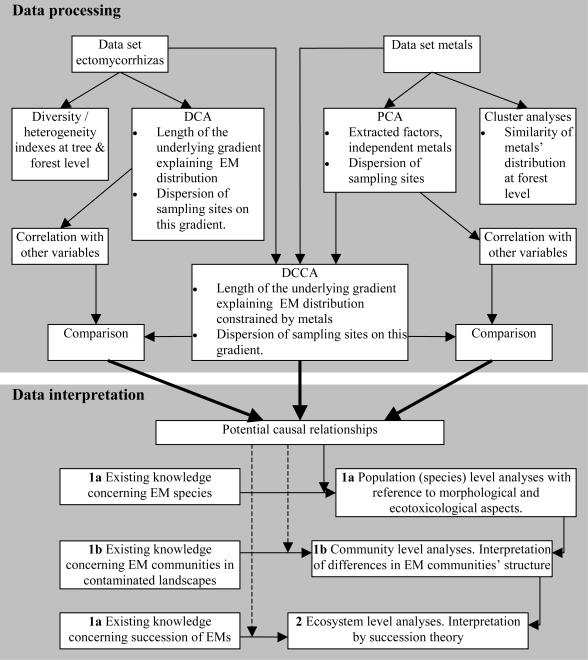
Schematic diagram showing the structure of data processing and interpretation in this article. Legend: DCA = detrended correspondence analyses, PCA = principal component analyses, DCCA = detrended canonical correspondence analyses.

In this case, we can present a structure of data processing and interpretation for analysis to give an example for the proposed ecological model (Figure 4). The relative abundance (i.e. numerical abundance of morphotypes) needed for computing the diversity indices has to be standardized to root length at α and β level (e.g., 20 cm), and to soil volume at γ level (by using the density of roots in a given soil volume). This will allow comparison within one study and also with other studies at γ level [63].

In order to extract all patterns from the raw data, for each sampled tree several indices (separately for upper and lower sampled layers) are to be computed. At tree level examples could be:

- the number of ECM species per tree
- the Berger-Parker index (maximum number of morphotypes on one tree species per total number of morphotypes)
- the coefficient of variation (CV) of species relative abundances (on standardized root length) around trees, and its average for all species present at a tree (AvCVSp)

At forest level, the indices could be:

- the average number of ECM species per tree and the derived coefficient of variation (CV = SD / average). The coefficient of variation applied at tree level is an indicator of the heterogeneity of distribution of ECMs in space around trees (in a given soil layer, upper or lower). At equal sampling effort around trees (e.g. four directions around a tree), a small CV indicates homogenous distribution of ECMs, and large ones heterogeneous distributions. The patterns of this CV are frequently correlated to disturbances of the ecosystem to which the trees belong.
- the average of the Berger Parker index and its CV
- coefficient of variation (CVa_t) around trees would reflect differences of trees with respect to the heterogeneity of ECM average of AvCVSp.
- the CV of ECM abundances between trees, for each species.
- coefficient of variation between trees to reflect differences of species distribution between trees (where a larger index shows higher species diversity between trees).

Comparison of diversity when the sampling effort is variable can be aided by the use of rarefaction techniques, which have been introduced in the study of ECM communities richness [64–67], and of diversity indices or similarities [68]. Rarefaction curves provide information on both aspects of diversity – richness and evenness [69] because the initial slopes is related to the Hulbert’s probability of interspecific encounter [70], which is a measure of evenness while the end-point indicates richness. However, not all rarefaction methods are equally appropriate. Poulin [71] found that the jackknife estimator and Chao’s estimator both improve the estimate of species richness, but they are imprecise and can seriously overshoot the true richness value when a community includes many rare species, and that the bootstrap estimator, on the other hand, gives a better estimate for species richness. Tedersoo *et al*. [66] reported the Chao2 estimator as less reliable than Jackknife2.

In order to extract relevant correlations, the number of control variables needs to be reduced (in case of the example of forests on metal contaminated lands, this would be relevant for bioavailable metal concentrations). Only a reduction will allow investigation of metal contamination with ECM distribution. Reduction of variables can be achieved with introduction of a contamination index [e.g., 72, 73], or by multivariate techniques, mainly principal component analyses (PCA) [e.g., 64, 74–77], or as independent variables for multivariate analyses of species data [78]. For PCA, the applicability should be verified, e.g. using Barttlet’s sphericity test, then a hierarchical cluster-analysis can be performed according to Ward’s method [79–82].

From the interpretation at population/species level it is possible to extract the mechanisms supporting higher level patterns. In case of ECM on contaminated lands, e.g., a cluster of species could be defined by Gherghel [62] which prevail under high As concentrations in the presence of higher P concentrations. In this case, this might be attributed to arsenate resistance conferred by the presence of phosphate. However, such predictions derived from interpretations at organism level need to be experimentally verified/falsified. As for the example given above with arsenic tolerance in phosphate-rich soils, there is proof. In the case of *Holcus lanatus*, *Agrostis capillaris* and *Deschampsia cespitosa*, AsO_4_^3−^ resistance is conferred by a suppression of the high affinity phosphate uptake system, since AsO_4_^3−^is a PO_4_^3−^ analogue and taken up by the PO_4_^3−^ uptake system [83]. Chen and Tibbet [84] demonstrated the ability of PO_4_^3−^to alleviate AsO_4_^3−^ toxicity for *S. variegatus* and *H. crustuliniforme*, both basidiomycetes. This was not the case, however, for *C. geophilum* [84]. These examples show, that correlations indicated by ecosystem level analyses can be verified for a derived function showing the applicability of our theoretical framework.

## 5. Conclusions

Based on the theoretical analyses of the literature we developed a concept framework for the investigation of ECM communities in secondary succession. The framework takes into consideration the relationship between the space-time scale of ECMs and that of the study area. In the case of a forest of several hectares this relation leads to considering for investigation a three levels hierarchy of systems. We could show that predictors can be derived at different levels of ecosystem structure and on different TDMs. This provides proof of concept for our theoretical framework, and is highly relevant for the interpretation of diversity patterns of ECM communities. It appears that is not appropriate to characterize the diversity of the ECM community in a forest as a single pool of organisms, and is more realistic to approach it at three hierarchical levels. Operationally, we used secondary succession in areas contaminated with multiple metals as an opportunity for testing the theoretical framework. Once the diversity patterns are appropriately described by a hierarchical ecosystem approach, it is possible to go back to the species level to explain these patterns by population and ecotoxicologic mechanisms.

## Figures and Tables

**Table 1.** Comparison of early- and late-stage ECM species characteristics.

Species/Characteristic	Reproduction	Genetic diversity	Requirement of C, N, P	Exploration types
Early	primarily by spores	higher	small	mainly medium and long distance
Late	primarily by clonal expansion	lower	greater	mainly contact and short distance
Source		Sarah *et al*. [50]		Our hypotheses
